# The Identification of Auxin Response Factors and Expression Analyses of Different Floral Development Stages in Roses

**DOI:** 10.3390/genes16010041

**Published:** 2025-01-01

**Authors:** Rui Huang, Xiaoni Zhang, Kaiqing Luo, Luke R. Tembrock, Sen Li, Zhiqiang Wu

**Affiliations:** 1 College of Horticulture, Shanxi Agricultural University, Jinzhong 030801, China; huang15565651712@163.com; 2 Shenzhen Branch, Guangdong Laboratory of Lingnan Modern Agriculture, Key Laboratory of Synthetic Biology, Ministry of Agriculture and Rural Affairs, Agricultural Genomics Institute at Shenzhen, Chinese Academy of Agricultural Sciences, Shenzhen 518120, China; zhangxiaoni@caas.cn (X.Z.); luokaiqing@caas.cn (K.L.); 3 Kunpeng Institute of Modern Agriculture at Foshan, Shenzhen Branch, Guangdong Laboratory of Lingnan Modern Agriculture, Agricultural Genomics Institute at Shenzhen, Chinese Academy of Agricultural Sciences, Shenzhen 518124, China; 4 Department of Agricultural Biology, Colorado State University, Fort Collins, CO 80523, USA; luke.tembrock@colostate.edu

**Keywords:** rose, auxin response factor, gene expression, flower development

## Abstract

**Background/Objectives:** *Auxin response factors* (*ARFs*) are important in plant growth and development, especially flower development. However, there is limited research on the comprehensive identification and characterization of *ARF* genes in roses. **Methods:** We employed bioinformatics tools to identify the *ARF* genes of roses. These genes were characterized for their phylogenetic relationships, chromosomal positions, conserved motifs, gene structures, and expression patterns. **Results:** In this study, a total of 17 *ARF* genes were identified in the genomes of *Rosa chinensis* ‘OB’, *R. chinensis* ‘CH’, *R. rugosa*, and *R. wichurana*. Based on RNA-seq analyses, we found that the *ARF* genes had diverse transcript patterns in various tissues and cultivars. In ‘CH’, the expression levels of *RcCH_ARFs* during different flower-development stages were classified into four clusters. In cluster 3 and cluster 4, *RcCH_ARFs* were specifically high and low in different stages of floral evocation. Gene expression and phylogenetic analyses showed that *RcCH_ARF3*, *RcCH_ARF4*, and *RcCH_ARF18* were likely to be the key genes for rose flower development. Conclusions: The identification and characterization of *ARF* genes in *Rosa* were investigated. The results presented here provide a theoretical basis for the molecular mechanisms of *ARF* genes in plant development and flowering for roses, with a broader application for other species in the rose family and for the development of novel cultivars.

## 1. Introduction

Auxin is a crucial phytohormone that has various effects on the regulators of plant growth and development [1]. There are several key auxin response genes, such as *auxin response factors*, that play central roles in the auxin response pathway of plants [2,3]. *ARFs* can mediate auxin transcriptional regulation by binding to auxin-responsive elements (TGTCTC, TGTCCC, TGTCAC, and TGTCGG) at the promoters of auxin-responsive genes [4,5]. *ARFs* typically contain the following three domains: a B3-like DNA-binding domain (DBD) at the N-terminus that specifically recognizes and binds the auxin response element; a middle domain that plays an activating or inhibiting role; and a C-terminus dimerization domain (CTD) that can bind directly to auxin/indole-3-acetic acid (*Aux*/*IAA*) [6,7]. *ARFs* participate in the auxin regulatory pathway in plants by binding to *Aux/IAA* [8]. When auxin concentrations are low, *ARFs* bind to *Aux/IAA* to form a dimer, which then binds to transcriptional repressors to prevent binding to target genes, thereby inhibiting the expression of auxin-responsive genes [9,10]. Conversely, when auxin concentrations are elevated, *ARFs* are released and the inhibitory effect is eliminated, leading to a series of auxin response processes [11,12].

*Arabidopsis thaliana* is the most extensively and comprehensively studied plant species in which the *ARF* gene family has been described and contains 23 *AtARF* members [13]. Researchers have elucidated the functions of *AtARFs*, providing detailed references for *ARF* gene family studies in other plant species. For example, *AtARF1* and *AtARF2* affect leaf senescence and floral organ shedding [14]. *AtARF3* regulates shoot apical meristem maintenance [15]. *AtARF19* and *AtARF7* control lateral root growth [16]. Furthermore, *ARFs* are also involved in flower organ growth: *arf6* and *arf8* mutants showed a slight delay in stem elongation and floral organ growth in *A. thaliana* [17]. *AtARF3* regulates the determinacy of the floral meristem [18]. *AtARF2–AtARF4* and *AtARF5* have essential roles in regulating both female and male gametophyte development [19]. Additionally, *ARF* genes have been comprehensively identified in various plants, including 23 in *Oryza sativa* [20], 31 in *Zea mays* [21], 22 in *Solanum lycopersicum* [22], 24 in *Cicer arietinum* [23], 20 in *Solanum melongena* [24], 13 in *Pinus koraiensis* [25], and 26 in *Dendrobium officinale* [26]. To date, there has not been a comprehensive study of *ARF* genes in roses; thus, it is necessary to identify and analyze *ARF* genes in roses to better understand their role in flowering.

Roses are one of the most famous flowers in the world, due in large part to their diverse, beautiful, and long-lasting flowers. Rose flowers are widely grown for use as cut flowers and as garden ornamentals, with extremely high esthetic and economic values. Auxin is a key hormone in rose development [27,28] and studies have demonstrated the essential role of auxin in the process of growth in roses. For instance, exogenous indole-3-acetic acid and naphthalene acetic acid affected the regeneration of damask-rose cuttings in three different growth media [29]. Auxin–cytokinin homeostasis is involved in the adventitious root formation of rose cuttings [30]. Petal abscission in fragrant roses is associated with auxin pathways [31]. As important genes in the auxin regulatory pathway, *ARFs* have been the subject of research regarding their function in the flower development of roses. For example, the *RhARF7–RhSUC2* module has been shown to be involved in the auxin regulation of SUC transport and, subsequently, regulates petal shedding [32]. Alternatively, *RhARF18* recruits *RhHDA6* to the promoter of *RhAG* to control petal–stamen transformation by enhancing inhibitory effects through histone deacetylation [33]. Furthermore, *RhARF2* regulates flower opening by controlling *RhMYB6* expression and by mediating the crosstalk between auxin and ethylene signaling [34]. However, the regulation of auxin on flower development in roses is minimal and the comprehensive identification of *ARF* genes in roses is still lacking.

With the completion of genome identification for several *Rosa* species and cultivars, it is possible to analyze genes that play an important role in flower growth and development. In this study, 17 *ARF* members were identified in four *Rosa* genomes. We analyzed the physical and chemical properties as well as the homology, conserved motifs, and gene structure for each gene. Furthermore, the expressions of these genes in different tissues and during different flower-development stages were investigated to understand the important roles of *ARFs* in *Rosa*. The results of this study provide a new foundation to further study the function of *ARFs* in rose flower development specifically as well as plant growth more generally.

## 2. Materials and Methods

### 2.1. Identification and Sequence Analysis of ARF Genes in Roses

The genomes of *R. chinensis* ‘Old Blush’ (hereafter ‘OB’) [35], *R. chinensis* ‘Chilong Hanzhu’ (hereafter ‘CH’) [36], *R. rugosa* [37], and *R. wichurana* [38] were downloaded from the Genome Database for Rosaceae (http://www.rosaceae.org/, accessed on 6 May 2024) and the National Center for Biotechnology Information (https://www.ncbi.nlm.nih.gov/, accessed on 6 May 2024) (accession number: PRJNA932466). The 23 ARF amino acid sequences of *A. thaliana* [13] and 25 ARF amino acid sequences of *O. sativa* [20] were obtained from The Arabidopsis Information Resource (TAIR; https://www.arabidopsis.org/, accessed on 6 May 2024) and the Rice Genome Annotation Project (RGAP; https://rice.uga.edu/, accessed on 6 May 2024) as query sequences, respectively. Then, the ARFs of the roses were identified as follows: first, ARF amino acid sequences from *A. thaliana* and *O. sativa* were used as queries to search for ARF genes in *R. chinensis* ‘OB’, *R. chinensis* ‘CH’, *R. rugosa*, and *R. wichurana* via a local BLASTP, with an E-value of ≤10^−5^. The sequences of ARFs were further confirmed by using the hidden Markov model (HMM) file (PF06507) from the Pfam database (http://pfam.xfam.org/search, accessed on 6 May 2024). The NCBI conserved domain database (https://www.ncbi.nlm.nih.gov/Structure/cdd/wrpsb.cgi, accessed on 6 May 2024) and SMART (https://smart.embl.de/, accessed 6 May 2024) were used to detect the amino acid sequences of candidate ARF members in *Rosa*. After identifying ARF members, ExPASy (http://web.expasy.org/protparam/, accessed on 7 May 2024) was used to analyze the physicochemical properties, such as the number of amino acids and molecular formulae. A subcellular localization prediction was conducted using Cell-PLoc 2.0 (http://www.csbio.sjtu.edu.cn/bioinf/plant-multi/, accessed on 7 May 2024).

### 2.2. Phylogenetic Analysis of ARFs in Roses

The full-length amino acid sequences of ARFs in *A. thaliana*, *O. sativa*, *Fragaria vesca* [39], and four *Rosa* cultivars were used to construct a phylogenetic tree by using the neighbor-joining method with 1000 iterations to obtain bootstrap values to assess the clade support. The phylogenetic tree was constructed using MEGA11 [40]. The tree was then visualized using the online tool Evolview v3 [41].

### 2.3. Chromosomal Location Analysis of ARFs

Using TBtools [42], we obtained the chromosome locations for each *ARF* gene according to the genome annotation file of the four *Rosa* cultivars.

### 2.4. Motifs and Gene Structure Analysis of ARFs

MEME (https://meme-suite.org/meme/tools/meme, accessed 12 May 2024) was used to identify the conserved motifs in the ARF amino acid sequences from the four *Rosa* cultivars using the patterns of 15 motifs. The gene structure information of the *ARFs* was confirmed using annotation data from the four genomes. TBtools was used to visualize the results.

### 2.5. Transcriptome Analysis of ARFs

To compare the expression patterns of *ARFs*, RNA-seq data from different tissues of *R. chinensis* ‘OB’ [43] and *R. chinensis* ‘CH’ [36] were downloaded. The transcriptome data of ‘CH’ samples during different flower-development stages were also used. The flower-development stages of ‘CH’ were divided into seven stages, including the vegetative meristem stage (DBO), the initiation stages of petals/petal-like structures and stamens/stamen-like structures (ISPS), the stage in which the hypanthium starts to sink below the perianth and stamens (HBPS), the early stage of flower bud formation (FBGP), the development of noncolored petals of flower buds (noncolored), the coloration of petals before flowering (coloring), and the emergence of fully colored petals during flower blooming (colored). Clean reads were obtained by removing low-quality reads and adapter sequences from the raw data. Using Hisat2 2.1.0 software, we mapped the clean reads to the reference genome [35,36]. The output of mapping was processed using String Tie to obtain FPKM (fragments per kilobase of exon model per million mapped fragments). The statistical analyses were conducted using GraphPad Prism and heat maps were drawn using TBtools.

## 3. Results

### 3.1. Identification of ARF Genes in Four Rosa Cultivars

To identify the *ARFs* of roses, local BLASTP and HMM searches were performed and domain detections were conducted. Finally, from each of four *Rosa* cultivars (*R. chinensis* ‘OB’, *R. chinensis* ‘CH’, *R. rugosa*, and *R. wichurana*), we identified 17 *ARF* members. The *ARFs* in the *Rosa* species were renamed based on homologous relationships with *F. vesca* (Table 1). Then, in order to more clearly understand the characteristics of *ARFs* in the *Rosa* species, the number of amino acids, molecular formula, prediction of molecular weight, theoretical isoelectric points, instability index, and prediction of subcellular localization were analyzed (Table S1). The number of amino acids of the ARFs ranged from 608 (RcOB_ARF17) to 1163 (RcOB_ARF7) in ‘OB’, 606 (RcCH_ARF17) to 1160 (RcCH_ARF7) in ‘CH’, 607 (RrARF17) to 1181 (RrARF7) in *R. rugosa*, and 569 (RwARF17) to 1165 (RwARF7) in *R. wichurana*. The predicted molecular weights for the ARF proteins ranged from 66.55 (RcOB_ARF17) to 130.21 (RcCH_ARF7), 66.36 (RcCH_ARF17) to 129.75 (RcCH_ARF7), 66.26 (RrARF17) to 1332.22 (RrARF7), and 62.68 (RwARF17) to 130.25 (RwARF7) kDa in ‘OB’, ‘CH’, *R. rugosa*, and *R. wichurana*, respectively. The theoretical isoelectric points varied from 5.22 (RcOB_ARF5) to 7.93 (RcOB_ARF18), 5.28 (RcCH_ARF5) to 7.92 (RcCH_ARF18), 5.21 (RrARF5) to 7.90 (RrARF18), and 5.22 (RwARF5) to 7.61 (RwARF18) in ‘OB’, ‘CH’, *R. rugosa*, and *R. wichurana*, respectively. The prediction of subcellular localization in the four *Rosa* cultivars showed that all genes were located in the nucleus.

### 3.2. Phylogenetic Analysis

**Figure 1:**
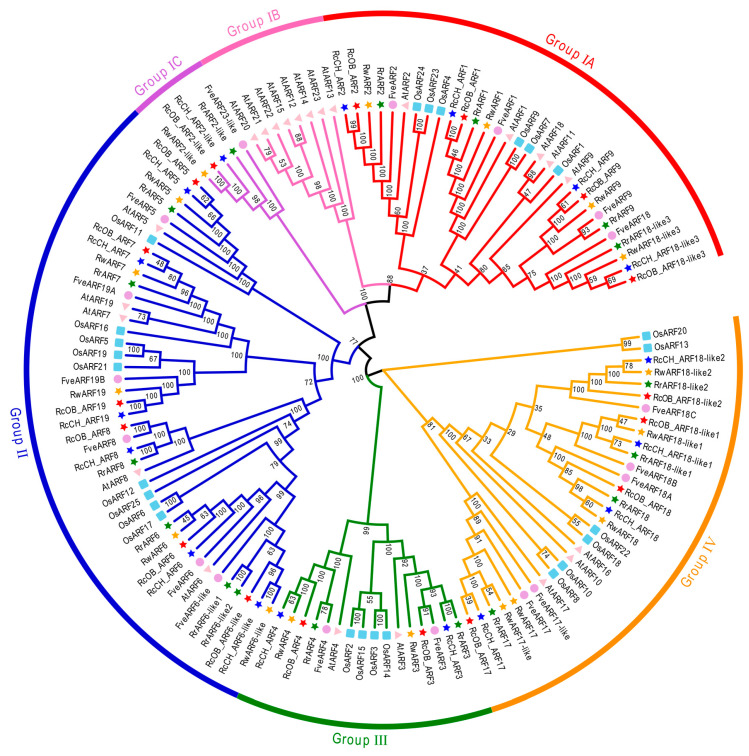
Phylogenetic tree of *ARFs*. Different-colored arcs represent different groups. Five stars of different colors represent different *Rosa* species (*R. chinensis* ‘OB’ in red, *R. chinensis* ‘CH’ in blue, *R. rugosa* in green, and *R. wichurana* in yellow). The lavender circles represent *F. vesca*, the light-pink triangles represent *A. thaliana*, and the light-blue squares represent *O. sativa*.

To analyze the evolutionary relationship of *ARF* genes in roses, a phylogenetic tree was constructed using 134 full-length amino acid sequences of ARFs from *A. thaliana*, *O. sativa*, *F. vesca*, and four *Rosa* cultivars (Figure 1). The 134 sequences were grouped into the following four major groups: Group I, Group II, Group III, and Group IV. Group I was further subdivided into Group IA, Group IB, and Group IC. Among these groups, Group II contained the greatest number of ARF genes in all four *Rosa* cultivars, which contained 6, 6, 6, and 5 ARFs in ‘OB’, ‘CH’, *R. rugosa*, and *R. wichurana*, respectively. Group III had the fewest members, with only two members in each cultivar of *Rosa*. A further analysis found that all ARFs of ‘OB’, ‘CH’, *R. rugosa*, *R. wichurana*, and *FveARFs* were on adjacent branches of the evolutionary tree, indicating the expected phylogenetic relationship of these genes as they are all species in the Rosaceae family.

### 3.3. Chromosomal Locations of ARFs in Rosa

**Figure 2:**
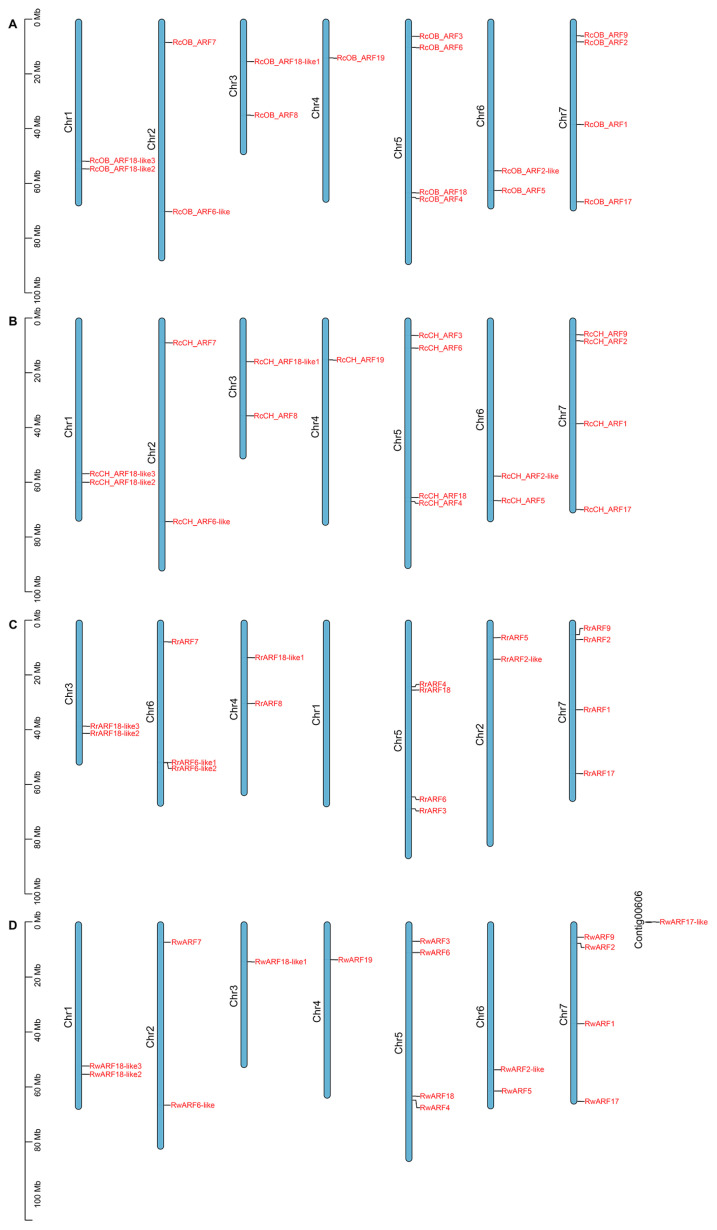
Chromosomal locations of *ARF* genes in four *Rosa* cultivars. (**A**) Chromosome location of *RcOB_ARFs*. (**B**) Chromosome location of *RcCH_ARFs*. (**C**) Chromosome location of *RrARFs*. (**D**) Chromosome location of *RwARFs*. Chromosome numbers are shown to the left of each chromosome. The scale on the left is chromosomal length in Mb.

We further investigated the chromosomal locations of *ARF* genes in *Rosa* (Figure 2). The analysis showed that *RcOB_ARFs* and *RcCH_ARFs* were unevenly distributed on 7 chromosomes, *RrARFs* were distributed across 6 of the 7 chromosomes, and *RwARFs* were distributed on 7 chromosomes and a contig. The largest number of *ARF* genes were found on chromosome 5 (Chr5) and Chr7, each with four genes. Chr4 of ‘OB’ and ‘CH’ contained the least number of *RcOB_ARFs* and *RcCH_ARFs*, with only one gene (comprising *RcOB_ARF19* and *RcCH_ARF19*). Chr3, Chr4, and Contig00606 of *R. wichurana* also contained only one gene, comprising *RwARF18-like1*, *RwARF9*, and *RwARF17-like*, respectively. Chr1 of *R. rugosa*, which was homologous with Chr4 of the other three *Rosa* cultivars, had no *ARF* genes. Moreover, Chr6 of *R. rugosa* had one more ARF than the homologous chromosomes of the other three *Rosa* cultivars, with three genes. A collinearity analysis showed that the *ARF* gene family of ‘OB’, ‘CH’, and *R. wichurana* had four pairs of segmental repeats, while *R. rugosa* had only three pairs of segmental repetitive genes (Figure S1).

### 3.4. Motifs and Gene Structure Analysis of the Four Rosa Cultivars

**Figure 3:**
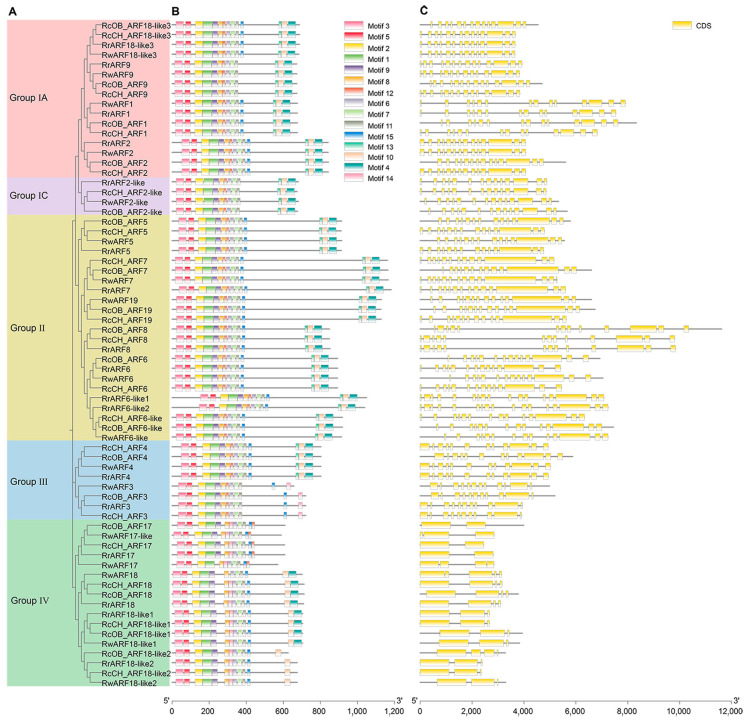
Phylogenetic tree, motifs, and gene structure of *ARF* genes in four *Rosa* cultivars. (**A**) Phylogenetic relationships of *ARF* genes from four rose cultivars. The pink-, purple-, yellow-, blue-, and green-colored blocks on the tree denote Group IA, Group IC, Group II, Group III, and Group IV, respectively. (**B**) Motif analyses of *ARFs*. The colored boxes, numbered 1–15, indicate different conserved protein motifs. (**C**) Gene structure of *ARF* genes. Exons are represented by yellow boxes; the lines between the boxes are introns.

To study the characteristic regions of ARF proteins in roses, 15 conserved motifs in rose ARF genes were identified using MEME and then analyzed in conjunction with the evolutionary tree (Figure 3A,B and Figure S2). Among them, motifs 2, 1, and 9 were identified as the B3-like DNA-binding domain (DBD) located at the N-terminus. Motifs 8, 12, 6, 7, and 11 represented the Auxin_resp domain. Motifs 10, 4, and 14 represented dimerization domains at the C-terminus (namely, CTD). The results showed that most *ARF* genes contained all three domains, while some members of Group III and Group IV only had the B3 and Auxin_resp domains and did not contain CTDs (*ARF3*, *ARF17*, *ARF17-like*, and *ARF18-like2*). The proportion of *ARF* members lacking the CTD was 17.6%, 17.6%, 17.6%, and 23.5% in ‘OB’, ‘CH’, *R. rugosa*, and *R. wichurana*, respectively. A further analysis found that the numbers and distribution of motifs were similar to their corresponding phylogenetic relationships. For example, the genes from Group I all had 13 or 14 motifs and were similar in structure. Similarly, the ARFs of Group II all contained 14 motifs and were similar in structure.

To explore the structural arrangement of *ARF* genes in roses, the exons and introns were analyzed (Figure 3C). The results showed that the distribution and number of exons in the *ARF* genes on the same branches were similar. For example, the *ARFs* from Group III contained 11 or 12 exons and their positions were similar. Additionally, the *ARF* genes in Group IV had the fewest exons, with only 2-5; their arrangements were also similar. The motifs and gene structure analyses both indicated that *ARFs* were highly conserved within a lineage, which helped us to further analyze the phylogenetic relationships and regulatory function of rose *ARFs*.

### 3.5. Expression of ARFs in Roses

**Figure 4:**
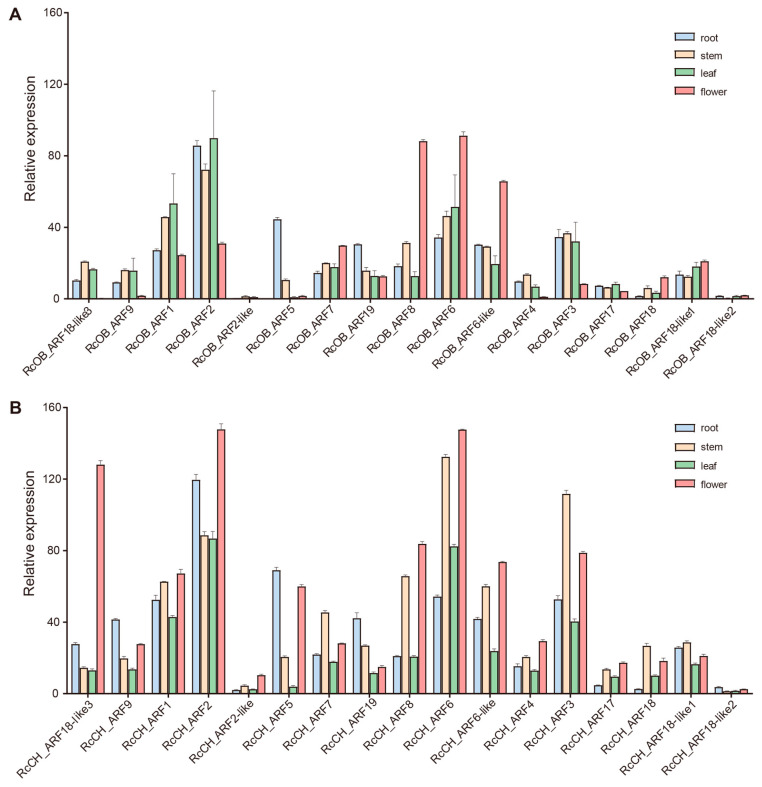
*RcARF* gene expression patterns in different tissues. (**A**) *RcOB_ARFs* expression in different tissues. (**B**) *RcCH_ARFs* expression in different tissues. The bars indicate one SD. Expression levels are given in FPKM.

Studies have shown that *ARFs* are involved in the growth and development of roots, stems, leaves, and flowers [6,7,44]. In order to better understand the potential function of *ARFs* in different tissues of roses, the expression levels of *ARFs* in different tissues of ‘OB’ and ‘CH’ were analyzed (Figure 4). We found that there were differences in the expression of *ARFs* between the two cultivars of roses; some genes showed a tissue-specific high expression, which may have been related to their function. For example, in ‘OB’, *RcOB_ARF5* was particularly highly expressed in the roots compared with the other tissues. However, in ‘CH’, the expression level of the homologous gene *RcCH_ARF5* was not only higher in the roots than in the other tissues, but also the expression level was significantly higher in the flowers than in the stems and leaves. Furthermore, in ‘CH’, *RcCH_ARF3* had a higher expression level in the stems compared with the other three tissues. However, in ‘OB’, the expression in the roots, stems, and leaves of homologous gene *RcOB_ARF3* was similar and was higher in all these organs than in the flowers. Additionally, the expression level of *RcOB_ARF8* in ‘OB’ was significantly higher in the flowers than in the other tissues and a similar pattern was observed for *RcOB_ARF6* and *RcOB_ARF6-like*. In ‘CH’, not only did the homologous genes *RcCH_ARF8*, *RcCH_ARF6*, and *RcCH_ARF6-like* possess similar expression patterns (as mentioned above), but also *RcCH_ARF18-like3* and *RcCH_ARF2* showed similar expression levels.

**Figure 5:**
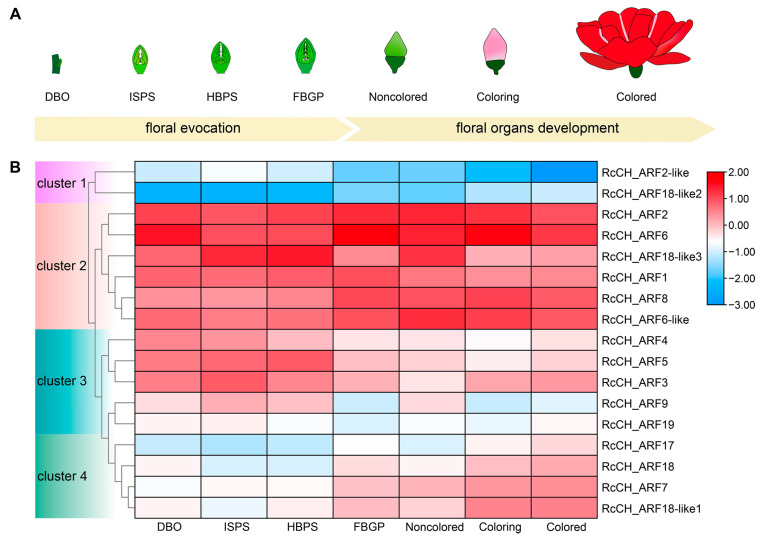
Expression of *RcCH_ARFs* during different stages of flower development. (**A**) Schematic diagram of different flower-development stages in *R. chinensis* ‘CH’. (**B**) Expression of *RcCH_ARFs* during different flower-development stages in *R. chinensis* ‘CH’. The results shown in the diagram are log2 (FPKM).

To more deeply investigate the role of *ARFs* in rose flower development, the expressions of *RcCH_ARF* genes during different stages of flower development were analyzed (Figure 5). The results showed that the expression patterns of *RcCH_ARFs* were classified into four clusters, which may be involved in different flower-development stages. The genes of cluster 1 had a low expression during all stages. The expression of *RcCH_ARFs* in cluster 2 were high throughout all flower-development stages. The expression of *RcCH_ARF* genes in cluster 3 and cluster 4 fluctuated during flower-development stages and showed similar expression trends, respectively. For instance, *RcCH_ARF4* in cluster 3 was highly expressed during the first three stages (DBO, ISPS, and HBPS), with a lower expression in later stages (FBGP, noncolored, coloring, and colored). Furthermore, the expression level of *RcCH_ARF18* in cluster 4 was low during the DBO–HBPS stages, but increased during FBGP through to the colored stage. Previous research has reported that *FveARF4* (homologous to *RcCH_ARF4*), *AtARF3* (homologous to *RcCH_ARF3*), and *FveARF18A* (homologous to *RcCH_ARF18*) are all involved in the regulation of flower development [18,39,45]. Thus, it could be inferred that *RcCH_ARF4*, *RcCH_ARF3*, and *RcCH_ARF18* may have similar regulatory roles during the stages of flower development.

## 4. Discussion

Auxin is an essential phytohormone involved in all stages of plant growth and development, including organogenesis, tissue differentiation, root initiation, fruit development, seed growth, and especially in the development of flowers [46,47,48,49]. These biological processes cannot be initiated without auxin. As important transcriptional factors, *ARFs* are involved in the auxin signal transduction pathway and play pivotal roles in regulating the expression of auxin-responsive genes [50,51]. For example, *FveARF2* can negatively regulate strawberry-fruit ripening and quality [52]. *FaARF2* mediates the receptacle ripening of strawberries via auxin–ABA interplay [53]. In addition, the *Mdm-miR160–MdARF17–MdWRKY33* module regulates cold stress tolerance by mediating ROS scavenging upon cold exposure in apples [54]. Auxin induces ethylene biosynthesis in apple fruit by activating the expression of *MdARF5*, which initiates apple-fruit ripening [55]. The positive feedback loop of *Mdm-miR160–MdARF17–MdHYL1* is necessary for apple survival under drought stress [56]. *PpARF6* plays a positive regulatory role in peach-fruit ripening by integrating auxin and ethylene signaling [57]. There are now numerous studies on fruit ripening and responses to stress, but research on the regulation of flower development by *ARFs* is relatively scarce.

Roses have become one of the most popular flowers in the world because of their rich color, fragrant aroma, and other desirable traits. Therefore, the analysis and characterization of *ARF* genes in *Rosa* species is necessary and can deepen our understanding of the role of *ARF* genes in rose development and aid in the development of new cultivars. In this study, based on the published genomes from four *Rosa* cultivars, a total of 17 *ARFs* were identified in ‘OB’, ‘CH’, *R. rugosa*, and *R. wichurana*, respectively. The genome sizes of these cultivars were 515 Mb [35], 541 Mb [36], 382 Mb [37], and 530 Mb [38] for ‘OB’, ‘CH’, *R. rugosa*, and *R. wichurana*, respectively. Among other plant genomes that have been sequenced and analyzed for *ARF* genes, *A. thaliana* (125 Mb) contains 23 *AtARFs* [13], *O. sativa* (466 Mb) contains *25 OsARFs* [20], *Z. mays* (2.3 Gb) contains 31 *ZmARFs* [21], and *Osmanthus fragrans* (727 Mb) contains 50 *OfARFs* [58]. The results above show that there is no association between the number of *ARF* genes and the genome size among different species.

The identification of conserved motifs is the foundation for studying gene functions. Our analysis of motifs found that *ARFs* on the same branch of the evolutionary tree had similar numbers and structural arrangements of motifs. Our results showed that all genes contained two structural domains, which were DBD and Auxin_resp domains. However, the CTDs of *ARF3*, *ARF17*, *ARF17-like*, and *ARF18-like2* were truncated. The proportion of *ARF* genes lacking the CTD in *Rosa* species (truncated *RcOB_ARFs* accounted for 17.6%, *RcCH_ARFs* for 17.6%, *RrARFs* for 17.6%, and *RwARFs* for 23.5%) were lower compared with other species such as *Medicago truncatula* (37.5%) [59], *Linum usitatissimum* (42.4%) [60], and *P. koraiensis* (38.5%) [25]. As such, it could be inferred that the CTD may be a key structural domain of *ARF* genes in *Rosa* species.

Phylogenetic trees are important methods used to resolve homologous relationships among various species or genes. In this study, we found that the *ARF* genes of the four *Rosa* cultivars we studied were most closely related to *FveARFs*, which reflected the taxonomic similarity of the lineages as they are both in the Rosaceae family. The function of *ARFs* has been extensively studied in various species, providing a valuable reference for exploring the potential functions of *Rosa ARF* genes. For example, the *FvemiR160–FveARF18A–FveAP1/FveFUL* module was verified to regulate the flowering time of woodland strawberries [39]. In *Rosa*, *RcOB_ARF18*, *RcCH_ARF18*, *RrARF18*, and *RwARF18* were genetically most similar to *FveARF18A*. Therefore, we could infer that *ARF18* of the *Rosa* species might play a similar role in regulating flower development. Additionally, the *miR390–tasiRNA3–ARF4* pathway delays the flowering time of woodland strawberries through *FveAP1/FveFUL* [45]. *ARF4* in the four *Rosa* cultivars was homologous to *FveARF4*, so it is reasonable to presume that *ARF4* of roses may function in a similar regulatory role in flower development. The function of *ARFs* in the model species *A. thaliana* has also been well-studied; for example, *AtARF3*, which controls floral meristem maintenance and termination by regulating cytokinin biosynthesis and signaling [61]. The *miR172/AP2* module regulates the size of the inflorescence meristem and *AP2* is regulated by *ARF3*, controlling the determination of the SAM size in *A. thaliana* [62]. Thus, *ARF3* in *Rosa* species may also regulate flower development. These comparisons of *Rosa ARFs* with other species provide an important set of hypotheses to test in future work.

Gene expression is an important criterion for determining if a gene is functioning in a given tissue or at time of development. In this study, we discovered that some genes showed flower-specific high expression; these were *RcOB_ARF8*, *RcOB_ARF6*, and *RcOB_ARF6-like* in ‘OB’ and *RcCH_ARF18-like3* in ‘CH’. We also found that *RcCH_ARF2* in ‘CH’ was not specifically highly expressed in flowers, but its expression level in flowers was higher than in other tissues. The expression patterns of *RcCH_ARF8*, *RcCH_ARF6*, *RcCH_ARF6-like*, and *RcCH_ARF4* in ‘CH’ were similar to *RcCH_ARF2*. Interestingly, of all the genes mentioned above in ‘CH’, *RcCH_ARF4* had high expression levels during the first three stages of floral evocation (DBO, ISPS, and HBPS), but the expression levels decreased during subsequent stages. In *F. vesca*, the function of the homologous gene *FveARF4* has been confirmed in flower development [45]. Therefore, *RcCH_ARF4* may have a downregulating role in the flower development of roses. This inference requires experiments to further demonstrate the mechanism of *RcCH_ARF4*. These results provide important data and well-resolved targets to help verify the function of *ARF* genes in regulating flower development. Knowing how *ARF* genes function in detail allows for the selection of desirable gene variants for the development of improved cultivars.

Research has reported the functions of *ARFs* in flower development [32,33,34], but there is still a limitation of genome-wide analyses and the characterization of *ARF* genes in roses. Moreover, research on the impact of *ARFs* on flower development in roses is scarce. Here, we conducted a comprehensive identification and bioinformatics analysis of *ARFs* in *Rosa*. Significantly, we identified *ARF* genes that may be involved in the regulation of flower development in roses; these were *RcCH_ARF3*, *RcCH_ARF4*, and *RcCH_ARF18.* From these analyses, this study aimed to comprehensively understand *ARF* genes in roses and reveal their roles in regulating flower development. These results provide information for the further functional analysis of *ARF* genes in roses, ultimately improving floral developmental traits in roses.

## 5. Conclusions

In this study, we performed a genome-wide identification of *ARF* genes in four *Rosa* cultivars, and a total of 17 *ARF* genes were identified in ‘OB’, ‘CH’, *R. rugosa*, and *R. wichurana*, respectively. These *ARF* genes were unevenly distributed on 7 chromosomes. An evolutionary analysis divided the *ARFs* of roses into four groups according to their homology with *AtARFs*, *OsARFs*, and *FveARFs*. The conserved motifs and gene structure analysis suggested that genes from the same lineage represented similar structures. The gene expression showed that *ARF* genes had different expression patterns in various tissues of ‘OB’ and ‘CH’. In ‘CH’, the expressions of *RcCH_ARFs* in cluster 3 and cluster 4 fluctuated during the flower-development stages, which were specifically high and low in the floral evocation stages, indicating that the *ARF* genes of roses may play essential roles in flower development. Importantly, *RcCH_ARF3*, *RcCH_ARF4*, and *RcCH_ARF18* were identified as highly likely to be regulatory factors in the flower development of roses. In short, this study provides valuable information for the functional verification of *ARFs* in rose flower development, which is applicable to future studies of functional genomics and the development of new cultivars.

## Figures and Tables

**Table 1:** Gene IDs and gene names of *ARFs* in *Rosa* cultivars.

Species	Gene Name	Gene ID
*R. chinensis* ‘OB’	*RcOB_ARF18-like3*	RcHm_v2.0_Chr1g0360041
	*RcOB_ARF9*	RcHm_v2.0_Chr7g0186081
	*RcOB_ARF1*	RcHm_v2.0_Chr7g0219771
	*RcOB_ARF2*	RcHm_v2.0_Chr7g0188911
	*RcOB_ARF2-like*	RcHm_v2.0_Chr6g0292551
	*RcOB_ARF5*	RcHm_v2.0_Chr6g0302551
	*RcOB_ARF7*	RcHm_v2.0_Chr2g0095551
	*RcOB_ARF19*	RcHm_v2.0_Chr4g0397771
	*RcOB_ARF8*	RcHm_v2.0_Chr3g0487771
	*RcOB_ARF6*	RcHm_v2.0_Chr5g0014961
	*RcOB_ARF6-like*	RcHm_v2.0_Chr2g0152951
	*RcOB_ARF4*	RcHm_v2.0_Chr5g0060011
	*RcOB_ARF3*	RcHm_v2.0_Chr5g0009381
	*RcOB_ARF17*	RcHm_v2.0_Chr7g0240691
	*RcOB_ARF18*	RcHm_v2.0_Chr5g0058761
	*RcOB_ARF18-like1*	RcHm_v2.0_Chr3g0469661
	*RcOB_ARF18-like2*	RcHm_v2.0_Chr1g0363241
*R. chinensis* ‘CH’	*RcCH_ARF18-like3*	evm.model.hB_v1.0_chr1.2384
	*RcCH_ARF9*	evm.model.hB_v1.0_chr7.760
	*RcCH_ARF1*	evm.model.hB_v1.0_chr7.3082
	*RcCH_ARF2*	evm.model.hB_v1.0_chr7.966
	*RcCH_ARF2-like*	evm.model.hB_v1.0_chr6.2903
	*RcCH_ARF5*	evm.model.hB_v1.0_chr6.3683
	*RcCH_ARF7*	evm.model.hB_v1.0_chr2.914
	*RcCH_ARF19*	evm.model.hB_v1.0_chr4.708
	*RcCH_ARF8*	evm.model.hB_v1.0_chr3.2842
	*RcCH_ARF6*	evm.model.hB_v1.0_chr5.1066
	*RcCH_ARF6-like*	evm.model.hB_v1.0_chr2.4774
	*RcCH_ARF4*	evm.model.hB_v1.0_chr5.3774
	*RcCH_ARF3*	evm.model.hB_v1.0_chr5.662
	*RcCH_ARF17*	evm.model.hB_v1.0_chr7.4543
	*RcCH_ARF18*	evm.model.hB_v1.0_chr5.3710
	*RcCH_ARF18-like1*	evm.model.hB_v1.0_chr3.1581
	*RcCH_ARF18-like2*	evm.model.hB_v1.0_chr1.2612
*R. rugosa*	*RrARF18-like3*	evm.model.Chr3.3276
	*RrARF9*	evm.model.Chr7.749
	*RrARF1*	evm.model.Chr7.3607
	*RrARF2*	evm.model.Chr7.969
	*RrARF2-like*	evm.model.Chr2.1948
	*RrARF5*	evm.model.Chr2.923
	*RrARF7*	evm.model.Chr6.1049
	*RrARF8*	evm.model.Chr4.3418
	*RrARF6*	evm.model.Chr5.5782
	*RrARF6-like1*	evm.model.Chr6.5248
	*RrARF6-like2*	evm.model.Chr6.5260
	*RrARF4*	evm.model.Chr5.2304
	*RrARF3*	evm.model.Chr5.6284
	*RrARF17*	evm.model.Chr7.5673
	*RrARF18*	evm.model.Chr5.2402
	*RrARF18-like1*	evm.model.Chr4.1801
	*RrARF18-like2*	evm.model.Chr3.3560
*R. wichurana*	*RwARF18-like3*	Rw1G025590.1
	*RwARF9*	Rw7G006530.1
	*RwARF1*	Rw7G027920.1
	*RwARF2*	Rw7G008560.1
	*RwARF2-like*	Rw6G029710.1
	*RwARF5*	Rw6G037050.1
	*RwARF7*	Rw2G007600.1
	*RwARF19*	Rw4G006600.1
	*RwARF6*	Rw5G010120.1
	*RwARF6-like*	Rw2G040900.1
	*RwARF4*	Rw5G036880.1
	*RwARF3*	Rw5G006830.1
	*RwARF17-like*	Rw0G013480.1
	*RwARF17*	Rw7G041690.1
	*RwARF18*	Rw5G036140.1
	*RwARF18-like1*	Rw3G014400.1
	*RwARF18-like2*	Rw1G027880.1

## Data Availability

The data presented in this study are available in the article and Supplementary Materials.

## Supplementary Materials

The following supporting information can be downloaded at https://www.mdpi.com/article/10.3390/genes16010041/s1. Figure S1: The chromosome distribution and interchromosomal relationships of *ARF* genes in four *Rosa* cultivars. (A) The chromosome distribution and interchromosomal relationships of *RcOB_ARFs* in ‘OB’. (B) The chromosome distribution and interchromosomal relationships of *RcCH_ARFs* in ‘CH’. (C) The chromosome distribution and interchromosomal relationships of *RrARFs* in *R. rugosa*. (D) The chromosome distribution and interchromosomal relationships of *RwARFs* in *R. wichurana*. Figure S2: The sequence logos of 15 conserved protein motifs in ARF genes of four *Rosa* cultivars. Table S1: Physicochemical properties of ARFs in four *Rosa* species.
