# Supplementary figures and images for: The Identification of Auxin Response Factors and Expression Analyses of Different Floral Development Stages in Roses
Source: Genes (Basel). 2025 Jan 1;16(1):41. doi: 10.3390/genes16010041 (PMC11764539; doi:10.3390/genes16010041)

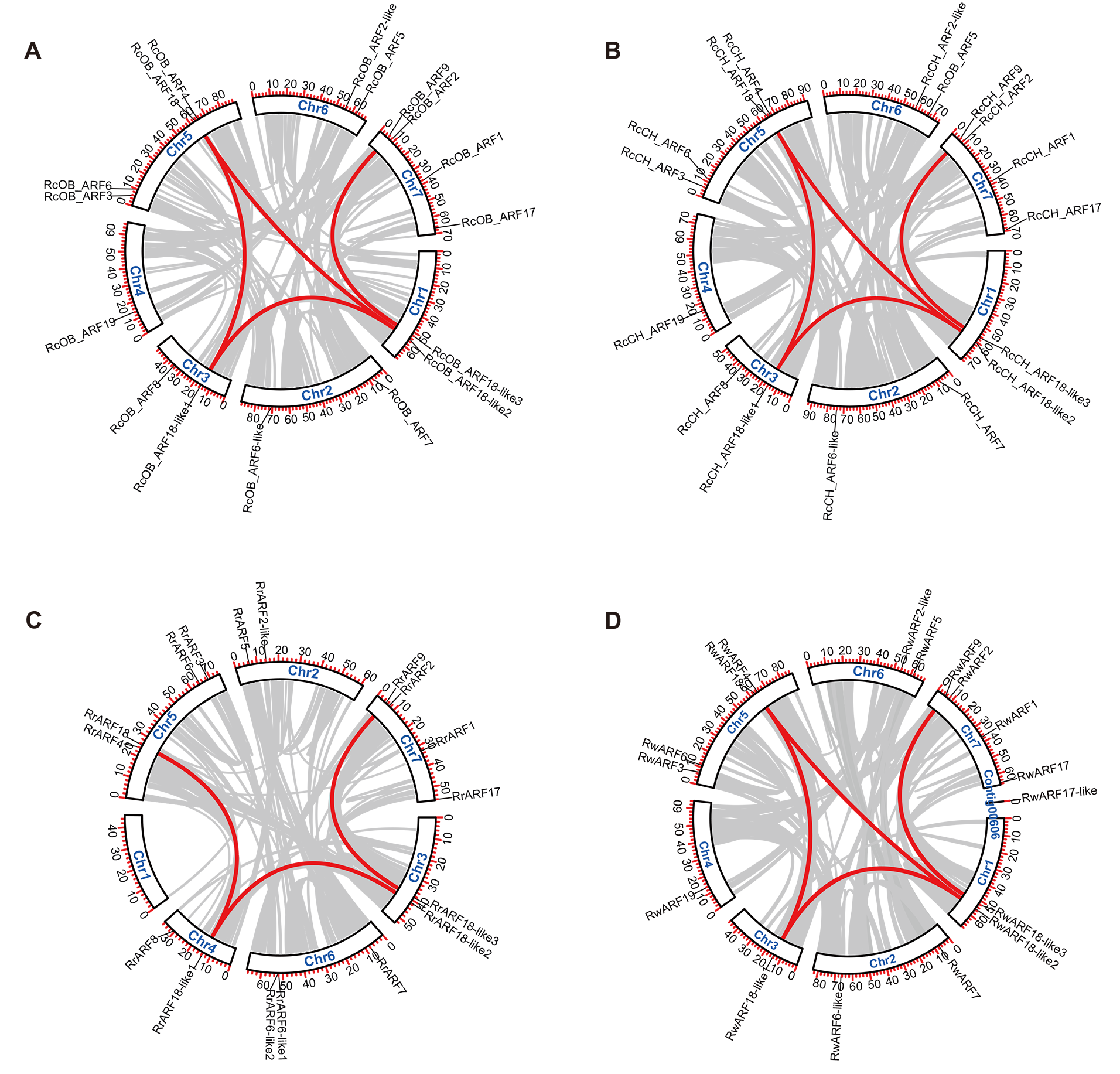

Supplement: Supplementary file 1 [file genes-16-00041-s001.zip › Figure S1.tif]

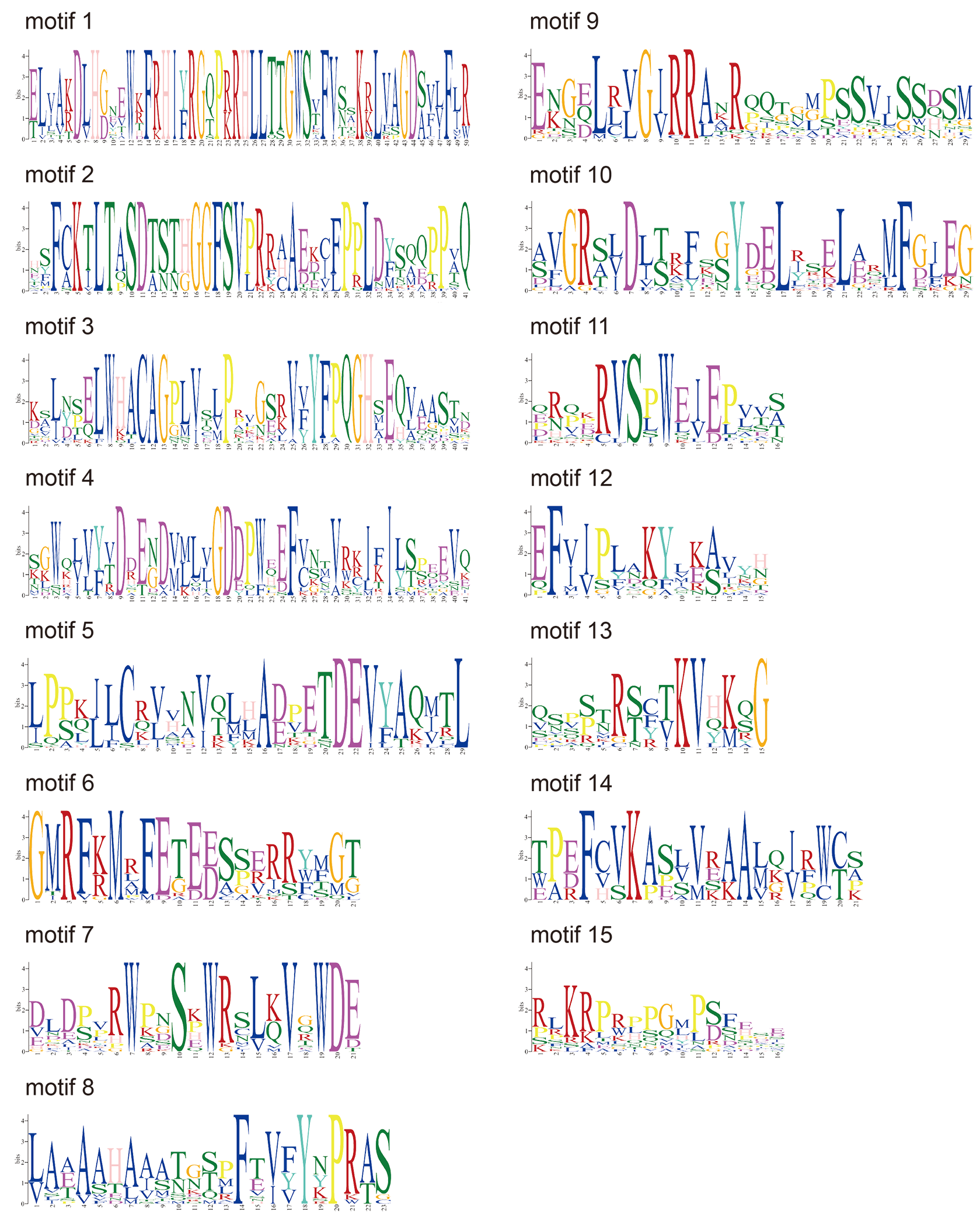

Supplement: Supplementary file 1 [file genes-16-00041-s001.zip › Figure S2.tif]
